# The Effect of Acute High-Intensity Interval Training on Executive Function: A Systematic Review

**DOI:** 10.3390/ijerph18073593

**Published:** 2021-03-30

**Authors:** Jing-Yi Ai, Feng-Tzu Chen, Shu-Shih Hsieh, Shih-Chun Kao, Ai-Guo Chen, Tsung-Min Hung, Yu-Kai Chang

**Affiliations:** 1 Department of Physical Education, National Taiwan Normal University, Taipei 106209, Taiwan; aijingyi@foxmail.com; 2 Sport Neuroscience Division, Advanced Research Initiative for Human High Performance (ARIHHP), Faculty of Health and Sport Sciences, University of Tsukuba, Ibaraki 3058577, Japan; alexnewtaipei@gmail.com; 3 Department of Psychology, Northeastern University, Boston, MA 02115, USA; stonehsieh79218@gmail.com; 4 Department of Health and Kinesiology, Purdue University, West Lafayette, IN 47907, USA; kao28@purdue.edu; 5 College of Physical Education, Yangzhou University, Yangzhou 225009, China; 6 Institute for Research Excellence in Learning Science, National Taiwan Normal University, Taipei 106209, Taiwan

**Keywords:** acute exercise, cognitive function, high-intensity interval training, executive function, exercise, systematic review

## Abstract

Acute high-intensity interval training (HIIT) is a time-efficient strategy to improve physical health; however, the effect of acute HIIT on executive function (EF) is unclear. The aim of this study was to systematically review the existing evidence and quantify the effect of acute HIIT on overall EF and the factors affecting the relationship between acute HIIT and EF. Standard databases (i.e., the PubMed, Medline, Scopus, and CENTRAL databases) were searched for studies that examined the effect of acute HIIT on EF and were published up until January 2021. The overall EF and factors grouped by three categories, namely, EF assessment characteristics, exercise intervention characteristics, and sample and study characteristics, were analyzed by percentage of comparison for positive or null/negative effects. Overall, 35 of 57 outcomes (61%) across 24 studies revealed that acute HIIT has a positive effect on overall EF. In terms of factors, the results indicated that among EF assessment characteristics, groups, inhibition, updating, and the assessment occurring within 30 min may moderate the effect of acute HIIT on EF, while among exercise intervention characteristics, total time within 11 to 30 min may moderate the effect. Finally, among sample characteristics, age under 40 years may moderate the effect. Acute HIIT is generally considered a viable alternative for eliciting EF gains, with factors related to EF components, timing of the assessment, exercise total time, and age potentially moderating the effect of HIIT on EF.

## 1. Introduction

Acute exercise, which refers here to a single bout of exercise, has received considerable and increasing interest due to its facilitating effect on cognitive function [1,2,3]. Indeed, it is currently recommended by both the American College of Sport Medicine guidelines (ACSM, 2020) and various national physical activity guidelines based in part on its beneficial effects on cognitive function [4]. Along with being positively associated with various aspects of cognitive function (e.g., attention, information processing, and memory), acute exercise has been found to have a pronounced effect on executive function (EF), a subset of top-down cognitive control processes for purposeful and goal-directed behavior [5,6,7,8]. Acute exercise has been observed to improve EF performance behaviorally [9,10,11], in addition to positively affecting EF task-associated neuroelectric activity [12,13,14] and neural networks [15,16]. The facilitation of EF following acute exercise has been further confirmed by several reviews [17,18,19]. However, such effects on specific modality of exercise (e.g., high-intensity interval training (HIIT)) remains for further investigation.

HIIT, a unique form of exercise, has been found to improve health in general [4]. HIIT consists of repeated, brief durations of high-intensity exercise (i.e., exercise resulting in ≥85% maximal heart rate) interspersed with periods of low-intensity exercise or rest [20,21,22]. Compared to typical exercise (e.g., aerobic exercise, resistance exercise), HIIT appears to constitute a more effective strategy for eliciting a range of physiological health benefits such as improved cardiorespiratory fitness [23,24], improved insulin sensitivity [25], and decreased body fat and blood pressure [26]. In addition to these physiological alterations, the beneficial effects of HIIT have also been found to extend to EF [27,28,29].

Recently, Chang and colleagues [30] proposed a “3W1H” framework aimed at further understanding the effects of acute exercise on cognitive function, in which it is suggested that the nature of cognitive functions and the exercise characteristics (i.e., what), moderators linked to sample characteristics and the timing of the assessment (i.e., who and when), and the potential mechanisms involved (i.e., how) should be considered. Relatedly, it is expected that the effects of acute HIIT on EF could be further enhanced based upon the framework. In specific terms, EF can be divided into two sub-domains consisting of core EF (i.e., inhibition, shifting, and updating/working memory) and higher-order EF (i.e., planning) [7,31], and the question of whether HIIT affects general EF or these specific EF sub-domains remains unanswered [19,32]. It is also unclear whether specific HIIT characteristics affecting EF could be investigated because while the nature of typical exercise characteristics (e.g., exercise duration, exercise mode, and exercise intensity) have been observed to affect EF [1,33], HIIT contains different exercise features (i.e., working/recovery time ratio and rest interval). Lastly, sample and study characteristics (i.e., age, gender, fitness level, design, and comparison) as well as the timing of the assessment have been found to influence the effects of acute exercise on cognitive function, but whether these factors affect the relationship between HIIT and EF has yet to be determined [1].

To extend the existing knowledge regarding the effect of acute HIIT on EF, systematic evaluations of the available evidence [27,28,29] and comprehensive reviews of the consequences of overall and moderating factors are required [34,35]. The present systematic review thus sought to investigate the effect of acute HIIT on EF while also considering three categories of potentially influential factors, including EF assessment characteristics (i.e., EF components and the timing of the assessment), exercise intervention characteristics (i.e., total time, type, modality, work/recovery time ratio, rest interval, and intensity), and sample and study characteristics (i.e., age, gender, fitness level, study design, and comparator), in order to further our understanding.

## 2. Methods

This systematic review was designed in accordance with the Preferred Reporting Items for Systematic Reviews and Meta-analysis (PRISMA) guidelines [36], and the methods used were selected based on the Cochrane Guidelines for Systematic Reviews for literature search and selection [37].

### 2.1. Search Strategy

The literature search consisted of a computer-based search of the PubMed, Medline, Scopus, and CENTRAL databases for articles published between January 1990 and January 2021. The search query was [“high intensity interval training” (Title/Abstract) OR “HIIT” (Title/Abstract) OR “high intensity interval exercise” (Title/Abstract) OR “HIIE” (Title/Abstract)] AND [“cognition” (Title/Abstract) OR “cognitive function” (Title/Abstract) OR “executive function” (Title/Abstract) OR “inhibition” (Title/Abstract) OR “updating” (Title/Abstract) OR “shifting” (Title/Abstract) OR “planning” (Title/Abstract)]. Additional articles were added by reviewing previous systematic reviews [24,27], and we also supplemented the search using complementary databases (i.e., the Google Scholar database). All the articles considered in the search were limited to peer-reviewed publications written in English.

### 2.2. Selection Criteria

The selection criteria were based on the PICOS criteria in order to define the characteristics of the included studies. Population: studies including healthy participants with no limitations for age were included; Intervention: studies evaluating the effect of acute HIIT on executive function and providing clear HIIT protocol were included; Comparator: studies comparing an active control (e.g., stretching) or passive control (e.g., watching television, sitting, and resting) were included; Outcomes: studies assessing EF were included; Study design: studies with any type of design (i.e., within-subject and between-subject designs) were included. One reviewer (JYA) initially performed the article search, after which two reviewers (JYA and FTC) screened the titles and abstracts of studies identified for potential selection by the search. Any disagreements were discussed with the third reviewer (YKC), until a consensus was achieved.

### 2.3. Quality Assessment

The Cochrane Collaborations’ domain-based assessment of risk of bias [38], a seven-item quality assessment tool, was employed. The seven items assessed can be described as follows: (1) random sequence generation refers to selection bias due to inadequate generation of a randomized sequence. (2) Allocation concealment refers to selection bias due to inadequate concealment of allocations prior to assignment. (3) Blinding refers to performance bias. (4) Blinding of the outcome assessment refers to detection bias. (5) Incomplete outcome data refers to attrition bias. (6) Selective reporting refers to reporting bias. (7) Other bias refers to bias due to problems not covered elsewhere in the domain-based assessment (e.g., a potential source of bias related to the specific study design). The methodological quality of each study was evaluated by two reviewers (JYA and FTC), and disagreements were resolved by consulting the third reviewer (YKC).

### 2.4. Data Extraction

Information on study details (i.e., author, year, and participant location), participants (i.e., sample size, gender, mean age, and fitness level), design characteristics (i.e., experimental design and comparator), exercise intervention (total time, type (modality), protocol (i.e., set, work and recovery time, and rest interval), and intensity), and the EF assessment (i.e., timing of the assessment, task, and components) were extracted from all the included studies. Data from included studies were extracted independently by two of the reviewers (JYA and FTC) and any discrepancies were solved by consulting a third reviewer (YKC).

For understanding potential moderators, the review further utilized the percentages of positive and null/negative effects. EF outcomes were extracted and response time (RT) data was included, if RT and accuracy were simultaneously coexisting [39]. We further categorized potential moderators into three groups to examine their possible moderating role in the effects of acute HIIT on EF (with the three categories being EF assessment characteristics, exercise intervention characteristics, and sample and study characteristics). The EF assessment characteristics considered included EF components (i.e., inhibition, updating, shifting, and planning) and the timing of the assessment (i.e., ≤10 min, 11–20 min, 21–30 min, or >30 min after the exercise). The exercise intervention characteristics considered included the total exercise time (i.e., ≤10 min, 11–20 min, 21–30 min, or >30 min), type (i.e., aerobic exercise or combined exercise), modality (i.e., running, cycling, circuit training, or boxing), rest interval (i.e., active or passive), work/recovery time ratio (i.e., >1, <1, or = 1), and intensity (i.e., maximal or submaximal). The sample and study characteristics considered consisted of age (i.e., ≤18 years old, 19~40 years old, or >40 years old), gender (i.e., >50% male, <50% male, or equal), fitness level (i.e., fit or sedentary), study design (i.e., within-subject or between-subject), and comparator (i.e., active control or passive control).

## 3. Results

### 3.1. Search Results and Overall EF

**Figure 1:**
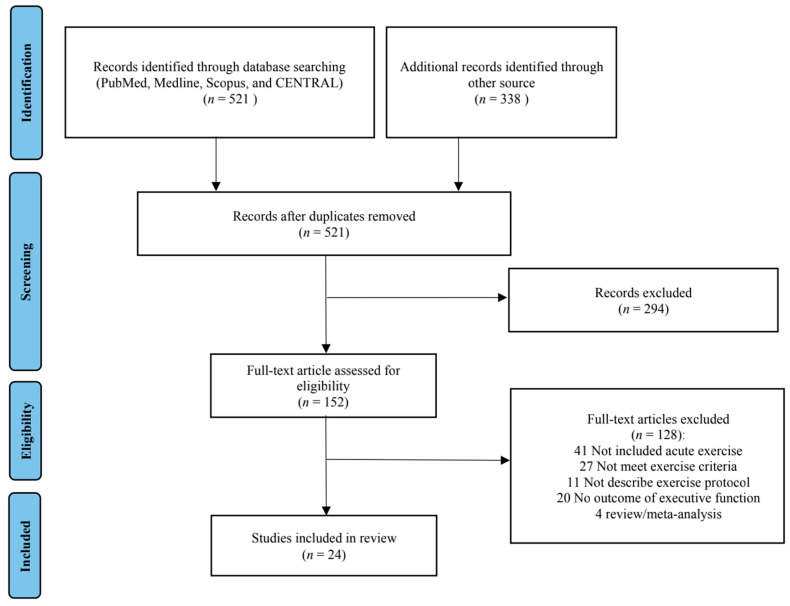
Preferred Reporting Items for Systematic Reviews and Meta-Analysis (PRISMA) flow diagram of each stage of the study selection.

A total of 521 articles for potential inclusion were initially retrieved through the search, and 338 articles were identified through other sources (*n* = 859). After removing duplicates, 446 articles remained for further screening of titles and abstracts. Afterward, 152 full-text articles were assessed for examination of their eligibility. Finally, 24 articles were included in the systematic review (Figure 1).

All of the included articles were studies addressing the effects of an acute HIIT intervention on EF; an overview of the included studies is provided in Table 1. The present review found that the number of participants in each study ranged from 11 to 64, and the studies included studies conducted in twelve countries, including Brazil [40], China [41,42], Colombia [43], France [44], Germany [45,46], Japan [47,48,49,50,51], Korea [52], Lithuania [53], Poland [54,55], Spain [56], Switzerland [57], the United States [58,59,60,61], and the United Kingdom [62,63]. Additionally, 13 task-related EF components (i.e., inhibition, updating, shifting, and planning) were categorized (Table 2). Overall, the results found that 35 of 57 (61%) outcomes showed a positive effect on overall EF and 22 of 57 (39%) outcomes showed a null/negative effect on overall EF (Table 3).

### 3.2. EF Assessment

In terms of individual EF components, we found greater percentages of positive effects for inhibition (69% vs. 31%) and updating (55% vs. 45%), whereas the results revealed that the number of null/negative effects was greater than the number of positive effects for shifting (29% vs. 71%). No EF outcomes for planning were included in the reviewed studies. In the terms of the timing of assessment, the number of positive effects was greater than the number of null/negative effects when the assessment was performed less than 10 min (67% vs. 33%), 11–20 min (88% vs. 12%), or 21–30 min (70% vs. 30%) after the exercise. In contrast, the number of null/negative effects was greater than the number of positive effects when the assessment was conducted more than 30 min (25% vs. 75%) after the exercise.

### 3.3. Exercise Intervention

This systematic review also examined the effects of acute exercise on EF through consideration of six exercise intervention characteristics, including typical exercise features (i.e., total time, type, and modality) and HIIT features (i.e., rest interval, work/recovery time ratio, and intensity).

Regarding typical exercise features, the analysis indicated that greater percentages of positive effects were found for total time between 11 and 20 min (71% vs. 29%) and total time between 21 and 30 min (65% vs. 35%), but not for total time of less than 10 min (38% vs. 62%) or total time of more than 30 min (40% vs. 60%). Regarding exercise type, the percentage of positive effects was greater than that of null/negative effects for aerobic exercise (65% vs. 35%), while the percentages of positive effects and null/negative effects were similar for combined exercise (50% vs. 50%). In terms of exercise modality, the percentage of positive effects was greater than that of null/negative effects for running (58% vs. 42%), cycling (69% vs. 31%), and boxing (67% vs. 33%). In addition, similar percentages of positive and null/negative effects were found for circuit training (50% vs. 50%).

Regarding HIIT features, the percentage of positive effects was greater than the percentage of null/negative effects for active interval control (70% vs. 30%) and for passive control (52% vs. 48%) during the rest interval. In terms of work/recovery time ratio, the analysis indicated that the percentage of positive effects was greater than that of null/negative effects for all categories, including >1 (64% vs. 36%), <1 (54% vs. 46%), and = 1 (63% vs. 37%). In terms of intensity, the percentage of positive effects was greater than the percentage of null/negative effects for maximal intensity (57% vs. 43%) and submaximal intensity (64% vs. 36%).

### 3.4. Sample and Study Characteristics

This systematic review considered also another five factors, including sample characteristics (i.e., age, gender, and fitness level) and study characteristics (study design and comparison type).

Regarding the sample characteristics, the results for age indicated that the percentage of positive effects was greater than the percentage of null/negative effects for participants aged less than 18 years (54% vs. 46%) and participants aged 19 to 40 years old (68% vs. 32%), whereas the percentage of null/negative effects was greater than percentage of positive effects for participants aged more than 40 years old (25% vs. 75%). In terms of gender, regardless of the percentage of male participant, the percentage of positive effects was greater than that of null/negative effects (52~67% vs. 33~48%). In terms of fitness level, the percentage of positive effects was greater than that of null/negative effects for fit participants (62% vs. 38%), whereas the percentages were similar for sedentary participants (50% vs. 50%). In terms of study characteristics, the percentage of positive effects was greater than that of null/negative effects for within-subject designs (66% vs. 34%) and between-subject designs (53% vs. 47%). In terms of comparators, the percentage of positive effects was greater than that of null/negative effects for passive control (62% vs. 38%) and similar for active control (50% vs. 50%).

### 3.5. Quality Assessment

**Figure 2:**
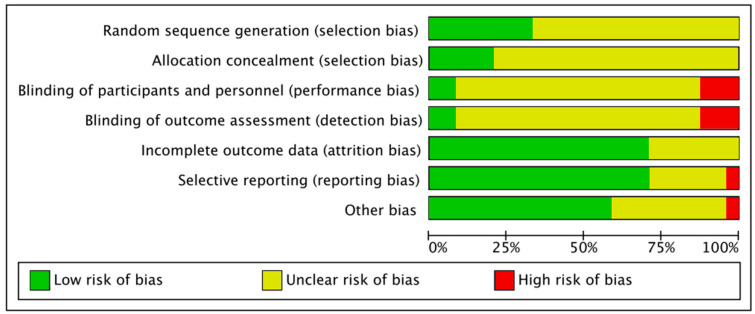
Domain-based assessments of risk of bias across studies based on the Cochrane Collaborations Handbook for Systematic Review.

Most of the studies revealed unclear risks of bias in terms of selection bias, performance bias, and detection bias, as well as low risks of bias in terms of attrition bias, reporting bias, and other bias. High risks of bias were reported in terms of performance bias, detection bias, reporting bias, and other bias, but with relative low percentages. These results demonstrated that the included studies were of low to moderate quality in general. The risk of bias results are summarized in Figure 2.

## 4. Discussion

This systematic review identified 57 outcomes across 24 articles in order to investigate the effect of acute HIIT on EF and to examine whether factors grouped into three categories, namely, EF assessment characteristics, exercise intervention characteristics, and sample and study characteristics, affect the relationship between HIIT and EF. Specifically, we used percentage of comparison (positive effects or null/negative effects) as an operational definition to identify the effect of acute HIIT on EF. Overall, the results revealed that acute HIIT generally tends to have a positive effect on EF. Moreover, they indicated that EF assessment characteristics (i.e., components, timing of the assessment), exercise intervention characteristics (i.e., total time) and sample characteristics (i.e., age) may all moderate the relationship between acute HIIT and EF.

### 4.1. Overall Effect

Sixty-one percent (35 of 57) outcomes from our analysis suggested that acute HIIT tends to have a positive effect on overall EF. The observed beneficial effect on EF effect of acute HIIT extends previous studies that utilized acute types of aerobic and resistance exercises [1] as well as high-intensity exercise [19] to determine their effects on EF. Remarkably, the results indicating the facilitation of EF following acute HIIT were provided by more updated, comprehensive (e.g., multiple exercise modality), and objective evaluations in this review compared to a previous systematic review [27].

The facilitation of EF possibly results from physiological alterations induced by HIIT (e.g., heart rate, lactate, catecholamine, and blood flow alterations), which in turn lead to an individual increasing his or her attentional sources when engaging in cognitive performances [64,65]. Previous studies have also provided evidence indicating that acute HIIT affects the prefrontal cortex, the brain region associated with EF, by increasing prefrontal cortex activation and oxygenation [59,63]. Furthermore, the observed EF facilitation might be associated with exercise-induced brain-derived neurotrophic factor (BDNF), a biomarker associated with EF [61,66]. Specifically, a single bout of HIIT induces higher brain H_2_O_2_ and TNF-α levels [67], after which these molecules activate the signaling of peroxisome proliferator-activated receptor-γ coactivator (PGC-1α) to enhance neuron BDNF synthesis [61,67]. It should be noted that these potential mechanisms have not been directly examined in terms of their role in the relationship between acute HIIT and EF, so more studies are needed to clarify their influence.

### 4.2. EF Assessment

Few studies have examined how acute HIIT affects EF from the perspectives of specific EF components and timing of the assessment. Our review reveals that acute HIIT tends to have positive effects on two specific components (i.e., inhibition and updating, but not shifting) and based on the timing of the assessment (i.e., within 30 min of the exercise but not more than 30 min after the exercise), implying that experimental variations affect the observed effects of acute HIIT on EF.

Most of the studies included in this review focused on inhibition (*n* = 39), and 69% of the outcomes indicated a positive effect of acute HIIT on EF. Inhibition is believed to reflect the ability to inhibit automatic responses when engaged in cognitive processes and has been observed to be positively linked to acute high-intensity exercise [68]. Given that inhibition was assessed by the studies in our review through multiple EF tasks including the Stroop test, the Flanker task, and the Go/No-Go task, our results suggest a positive effect of acute HIIT on inhibition regardless of the assessment task employed.

A total of 55% of outcomes indicated a positive effect of HIIT on updating, but only 29% of outcomes showed a positive effect on shifting, implying that acute HIIT has varying effects on specific EF components. Although updating and shifting are recognized as aspects of core EF, the concepts and involved brain regions of these two EF components seem to be different. Specifically, updating (also known as working memory), a capacity for processing and restoring temporary information [69,70], is associated with the dorsolateral prefrontal cortex of the middle frontal gyrus, while shifting (also known as cognitive flexibility), which refers to the ability to perceive alternative explanations for occurrences and to modify responses while overriding automatic behaviors [71], is associated with the anterior cingulate (located at anterior and posterior regions). Our findings suggest that acute HIIT may affect these two components differently. Notably, however, given that the numbers of outcomes regarding updating (*n* = 11) and shifting (*n* = 7) were relatively limited, the results should be interpreted with caution. Furthermore, it is noteworthy that there were no studies focused on planning. Planning is believed to reflect an ability to manage a strategy and set goals and is considered a higher-level and primary component of EF [7]. Our results therefore call for caution in examining the specific EF components to further our understating regarding the impacts of acute HIIT on EF.

In terms of timing of the assessment, our findings indicate that positive effects of acute HIIT on EF were observed for assessments conducted within 30 min of the exercise (i.e., ≤10 min, 11–20 min, 21–30 min) but not for assessments conducted more than 30 min after the termination of the exercise suggest that the beneficial effects of acute HIIT on EF were diminished after 30 min. The finding of decreased effects on EF with longer time following acute exercise is partially supported by a previously conducted meta-analysis [1], in which larger positive effects on cognitive function were observed within 11 to 20 min following acute exercise (effect size (ES) = 0.26) and smaller effects were showed after 20 min (ES = 0.17). Indeed, Cooper, Bandelow, Nute, Dring, Stannard, Morris and Nevill [62] hypothesized that the attenuated magnitude of the improvement of cognitive performance at 30 min following the cessation of HIIT is likely associated with higher neuromuscular fatigue during exercise at 95% of maximal power output. It is thus possible that the beneficial effect on EF would be diminished along with the passing of time following the termination of acute HIIT.

### 4.3. Exercise Intervention

The present review included past studies that investigated both typical exercise characteristics (i.e., total time, type, and modality) and specific HIIT characteristics (i.e., rest interval, work/recovery time ratio, and intensity) in order to examine the role of these exercise characteristics in the relationship between acute HIIT and EF.

Acute HIIT interventions with total time between 11 and 20 min (17 of 24 outcomes, 71%) or between 21 and 30 min (13 of 20 outcomes, 65%) tended to have positive effects on EF, but those with total time of less than 10 min or more than 30 min did not consistently have positive effects on EF (38~40% for positive effects). These findings confirm previous empirical studies indicating that acute exercise durations of 11 to 30 min are required to increase EF. For example, Chang et al. [72] focused on younger adults to examine the dose–response relationship between acute exercise duration and EF and found that acute exercise for 30 min (i.e., 5 min warm-up, 20 min main exercise, and 5 min cool-down) has beneficial effects on EF, whereas acute exercise for 10 min or 45 min showed negligible effects. Their follow-up study focused on late-middle-aged adults further demonstrated that acute exercise bouts for 30 min show more enhanced EF compared to those of less than 20 min [73]. However, given that these studies focused on acute aerobic exercise, whether or not there is a dose–response relationship between HIIT duration and EF requires further examination.

Interestingly, HIIT types (i.e., aerobic exercise and combined exercise) and HIIT modality (i.e., running, cycling, and boxing) favor positive effects (i.e., 58 to 69%) on EF, wherein HIIT types (i.e., combined exercise) and HIIT modality (i.e., circuit training) show 50% outcomes favor positive effects. Along with a study by Hsieh, Chueh, Huang, Kao, Hillman, Chang, and Hung [27] indicating that HIIT with aerobic exercise type facilitates EF, our review extends previous findings by suggesting that HIIT type and modality generally have positive influences on EF.

Regarding rest interval, 70% positive outcomes for active rest (e.g., low-intensity exercise) and 52% positive outcomes for passive rest (i.e., full rest) were observed. The results suggest that while both rest types benefit EF, HIIT with active rest may lead to more positive effects. Indeed, the beneficial effects of acute HIIT with active rest were observed not only immediately after [63] but also at 60 min after acute HIIT [57]. Our review also examined the role of work/recovery time ratios categorized into “>1” (i.e., exercise time is greater than rest time), “<1” (i.e., rest time is greater than exercise time), and “=1” (i.e., exercise time and rest time are equal) and observed that all the categories showed greater positive percentages (i.e., from 54% to 64%). While Hsieh, Chueh, Huang, Kao, Hillman, Chang, and Hung [27] have suggested that a ratio of 1 is better than other ratios based upon a specific empirical study [57], our results from other objective evaluations demonstrate that the work/recovery time ratio of HIIT is not a sensitive factor in terms of the effects of HIIT on EF.

Our findings indicated that acute HIIT tends to have a positive effect on EF regardless of its intensity (i.e., submaximal or maximal), which is in contrast with previous hypotheses, such as the inverted-U theory [74], reticular-activation hypofrontality theory [75], and neurochemical hypotheses [64], which suggest that acute exercise of high intensity seems to impair EF. It is worth noting, however, that those hypotheses were proposed based upon aerobic exercises with continuous rhythms, which are different from HIIT containing numerous short bouts of high-intensity exercise and rest, suggesting that the special form of HIIT might result in different effects on EF.

### 4.4. Sample and Study Characteristics

Three individual sample characteristics including age, gender, and fitness level were analyzed. We categorized age so as to present the adolescents, adults, and middle-aged to older adults. We believe the ages would be considered as main moderators between HIIT and EF. Greater percentages of positive effects were found, for both populations under 18 years of age, and between 19 to 40 years of age (i.e., 54% and 68%, respectively), but not for those of more than 40 years of age (i.e., 25%). However, these results should be interpreted with caution, because the number of studies focusing on individuals older than 40 years was limited (*n* = 4), and none of the selected studies targeted older adults. The limited numbers of such studies may be linked to HIIT features, namely, the high-intensity and quick rhythm of HIIT, which raise safety issues and are a major exercise barrier for its (more) widespread implementation, particularly among older individuals [76]. Clearly, optimal HIIT in terms of duration, intensity, type, and modality, must be considered for middle-aged or older populations.

The results for all genders (i.e., >50% male, <50% male) and fitness levels (i.e., fit, sedentary) showed greater percentages of positive effect, with males and individuals with fit status showing higher positive percentages (62 to 67%). These findings suggest that acute HIIT might have both general and specific effects on EF in terms of these two individual characteristics.

Study characteristics were categorized into two dimensions (i.e., study design and comparator). More studies (*n* = 38) employed within-subject designs than between-subject designs (*n* = 19), which was consistent with previous studies regarding the effects of acute exercise on cognitive function indicating that cross-over designs are typical employed [1]. Although within-subject designs showed greater percentages of positive effects (i.e., 66%) compared to between-subject designs (i.e., 53%), the results of within-subject and between-subject study designs should be interpreted differently, given that a within-subject design has more power but potentially suffers from confounds, while a between-subject design is more conservative but has limitations in some cases [77]. We therefore suggest the choice of design should be further considered. Lastly, we also included both active and passive controls to confirm whether the observed effects of acute HIIT on EF were affected by comparator, and the results indicated that passive control is showed more effects on EF (62%) compared to active control (50%). This finding is important because it indicates that acute HIIT tends to have positive effects regardless of the comparator. However, further studies are needed to replicate these findings because only a small number of active controls were utilized (*n* = 2).

### 4.5. Strength and Limitations

This was first systematic review to have examined the effects of HIIT on EF with consideration of multiple factors, as well as considering evaluations by using objective approaches; however, it also had some limitations. The first was the small number of studies included overall. HIIT is a relatively new exercise modality and was initially utilized mainly to improve athletic performance [78]. Most studies of HIIT have thus focused on its effects on physical health [4,79], while the examination of its effects on mental health, including cognitive function, began relatively recently, which limited the number of studies included in this review. Additionally, for fully understanding the research issue, unpublished articles could be further considered, in order to reduce publication bias (e.g., file drawer problem). Secondly, only a few studies were judged as low risk, and the associated methodological restrictions increased the possibility that the results were swayed by confounding variables, meaning that caution should be used in drawing any conclusions. Furthermore, we excluded several studies (*n* = 11) because the details of HIIT intervention (e.g., work/recovery time, intensity) were missing. Future studies are suggested in order to provide competed information about HIIT intervention. Lastly, while the percentages of positive verses negative effects provide a relatively objective means for evaluating the results, statistical analysis of effect sizes, obtained via meta-analysis, provides more precise conclusions, and therefore is suggested for use in future studies.

## 5. Conclusions

The current systematic review indicated that the majority of HIIT analyses indicated positive effects of HIIT on EF, and that the beneficial effects on EF associated with acute HIIT occurred regardless of exercise type, modality, rest interval, work/recovery time ratio, intensity, gender, fitness level, and comparator, with various EF assessment characteristics (i.e., components, timing of the assessment), exercise intervention characteristics (i.e., total time), and sample characteristics (i.e., age) potentially moderating the observed relationship between acute HIIT and EF. We believe our findings have important applications for enhancing the effects of acute HIIT on EF, especially for those seeking time-efficient designs of HIIT interventions to immediately facilitate EF.

## Figures and Tables

**Table 1:** Overview of characteristics of included studies regarding acute high-intensity interval training and executive function.

Study	Participant	Design	Exercise Intervention				EF Assessment		
Author (year), Location	N (males %) Mean age (SD) Fitness level	Exp. Design Comparator	Total time	Type	Protocol: Set, WRT (rest interval)	Intensity	Time exam.	Task	Comp.
Alves et al. (2014) [40] Brazil	*N* = 22 (41%) 53.7 (4.7) Fit	Within-subject Active	20 min	Aerobic exercise (cycling)	10 sets, 1 min and 1 min (active)	Submax.	Immediate	Digit span test-backward Stroop test	Updating Inhibition
Burin.et al. (2020) [47] Japan	*N* = 45 (53%) 23.7 (4.5) Fit	Within-subject Passive	8 min	Aerobic exercise (running)	8 sets, 30 s and 30 s (active)	Maximal	Immediate	Stroop test	Inhibition
Chang et al. (2017) [52] Korea	*N* = 36 (0%) 21.4 (1.6) Fit	Between-subject Passive	30 min	Combined exercise (circuit training)	3 sets, 1:2 (passive)	Submax.	15 min	Stroop test	Inhibition
Cooper et al. (2016) [62] UK	*N* = 44 (48%) 12.6 (0.6) Fit	Within-subject Passive	10 min	Aerobic exercise (running)	10 sets, 10 s and 50 s (active)	Maximal	Immediate, 45 min	Corsi block test Stroop test-complex level	Updating Inhibition
Dupuy et al. (2018) [44] France	*N* = 20 (100%) 28.0 (4.8) Fit	Within-subject Passive	36 min	Aerobic exercise (cycling)	6 sets, 3 min and 3 min (passive)	Submax.	15, 30, 45, and 60 min	Modified Stroop test-interference	Shifting
Gmiat et al. (2017) [55] Poland	*N* = 14 (0%) 22.7 (3), 41.7 (4) Sedentary	Between-subject Passive	27 min	Combined exercise (circuit training)	3 sets, 30 s and 10 s (passive)	Maximal	60 min	Corsi block test Stroop test	Inhibition Updating
Hashimoto et al. (2018) [48] Japan	*N* = 14 (100%) 24 (1) Sedentary	Within-subject Passive	28 min	Aerobic exercise (cycling)	4 sets, 4 min and 3 min (active)	Submax.	Immediate, 10, 20, 30, 40, 50 min	Stroop test	Inhibition
Kao et al. (2018) [58] USA	*N* = 36 (50%) 21.5 (0.5) Sedentary	Within-subject Passive	16 min	Aerobic exercise (running)	8 sets, 1 min and 1 min (active)	Submax.	12 min	Flanker task-interference score	Inhibition
Kao et al. (2017) [59] USA	*N* = 64 (42%) 19.2 (0.8) Sedentary	Within-subject Passive	7.5 min	Aerobic exercise (running)	3 sets, 1.5 min and 1 min (active)	Submax.	20 min	Flanker task-incongruent	Inhibition
Kujach et al. (2018) [49] Japan	*N* = 25 (64%) 21.0 (1.6) Sedentary	Within-subject Passive	8 min	Aerobic exercise (cycling)	8 sets, 30 s and 30 s (passive)	Submax.	15 min	Stroop test	Inhibition
Kujach et al. (2019) [50] Japan	*N* = 36 (100%) 21 (1.29) Fit-low	Between-subject Passive	30 min	Aerobic exercise (cycling)	6 sets, 30 s and 4.5 min (passive)	Maximal	20 min	Stroop test TMT-B	Inhibition Shifting
Lambrick et al. (2016) [63] UK	*N* = 20 (45%) 8.8 (0.8) Sedentary	Within-subject Passive	15 min	Aerobic exercise (running)	6 sets, 55 s and 95 s (active)	Maximal	1 min, 15 min, and 30 min	Stroop test	Inhibition
Ligeza et al. (2018) [54] Poland	*N* = 18 (100%) 24.9 (2.2) Fit	Within-subject Passive	24 min	Aerobic exercise (cycling)	4 sets, 3 min, 3 min (active)	Submax.	13 min	Flanker task-incongruent	Inhibition
Ludyga et al. (2019) [57] Switzerland	*N* = 94 (100%) 13.9 (0.7) Sedentary	Between-subject Passive	16 min	Combined exercise (circuit training)	10 sets, 60 s and 30 s, 30 s and 30 s (passive)	Maximal	Immediate, 30 min, 60 min	Flanker task-incongruent	Inhibition
Martínez et al. (2020) [56] Spain	*N* = 25 (100%) (2.1) Fit	Within-subject Passive	20 min	Aerobic exercise (cycling)	10 sets, 1 min and 1 min (passive)	Submax.	Immediate, 30 min	Digit span test	Updating
Miller et al. (2019) [60] USA	*N* = 25 (48%) 23 (2.79) Fit	Within-subject Passive	10 min (LV) and 20 min (MV)	Aerobic exercise (cycling)	5 sets, 1 min and 1 min (active) (LV); 10 set, 1 min and 1 min (active) (MV)	Submax.	1 min	Stroop test	Inhibition
Quintero et al. (2018) [43] Colombia	*N* = 36 (100%) 23.55 (3.4) Sedentary	Between-subject Passive	32 min	Aerobic exercise (running)	4 sets, 4 min and 4 min (active)	Submax.	Immediate	Stroop test-interference	Inhibition
Schwarck et al. (2019) [45] Germany	*N* = 39 (100%) 23.33 (3.23) Fit	Between-subject Passive	25 min	Aerobic exercise (running)	5 sets, 2 min and 3 min (active)	Submax.	10 min	Stroop test-incongruent TMT-B	Inhibition Shifting
Slusher et al. (2018) [61] USA	*N* = 13 (100%) 23.62 (1.06) Sedentary	Within-subject Passive	5 min	Aerobic exercise (cycling)	10 sets, 20 s and 10 s. (active)	Maximal	Immediate	Wisconsin card sorting task	Updating
Solianik et al. (2020) [53] Lithuania	*N* = 11 (100%) 22.8 (2.9) Fit	Within-subject passive	12 min	Aerobic exercise (boxing)	3 sets, 3 min and 1 min (passive)	Maximal	21–30 min	Go/No-Go task Procedural reaction time Mathematical processing	Inhibition Shifting Updating
Sun et al. (2019) [42] China	*N* = 20 (50%) 23.9 (2.5) Sedentary	Within-subject Passive	6 min	Aerobic exercise (cycling)	10 sets, 6 s and 30 s (passive)	Maximal	Immediate	Go/No-Go task	Inhibition
Tsukamoto et al. (2016) [51] Japan	*N* = 12 (100%) 22.9 (0.4) Fit	Within-subject Passive	28 min	Aerobic exercise (cycling)	4 sets, 4 min and 3 min (active)	Submax.	Immediate, 10, 20, and 30 min	Stroop test-incongruent	Inhibition
Wilke et al. (2020) [46] Japan	*N* = 35 (49%) 26.7 (3.6) Fit	Between-subject Passive	15 min	Combined exercise (circuit training)	30 sets, 20 s and 10 s (passive)	Maximal	Immediate	Stroop TMT-B Digit span test	Inhibition Shifting Updating
Xie et al. (2020) [41] China	*N* = 16 (100%) 24.5 (5.09) Sedentary	Within-subject Passive	20 min	Aerobic exercise (cycling)	10 sets, 1 min and 1 min (active)	Submax.	15 min	Flanker task-incongruent	Inhibition

Note: WRT = work and recovery time; NR = not report; TMT-B = trail making test-B; UK = United Kingdom; USA = United States; Immediate ≤10 min; Exam. = Examination; Comp. = Component; Submax. = Submaximal.

**Table 2:** Overview of component-specific executive function tasks.

Inhibition	Updating	Shifting	Planning
Go/No-Go task Flanker task Stroop test	Corsi blocks test Digit span test-backward Mathematical processing Symbol digits modality Wisconsin card sorting task	Modified Stroop test Procedural reaction time Switching task-costs Trail making task-B	None

**Table 3:** The positive and null/negative comparison of effects on overall executive function and associated factors categorized into three groups.

	*N* of Outcome	*N* of Positive Effect (%)	*N* of Null Effect (%)
**Overall**	**57**	**35 (61%)**	**22 (39%)**
**EF assessment**			
Component			
Inhibition	39	27 (69%)	12 (31%)
Updating	11	6 (55%)	5 (45%)
Shifting	7	2 (29%)	5 (71%)
Planning	--	--	--
Time examination			
≤10 min	24	16 (67%)	8 (33%)
11–20 min	11	9 (88%)	2 (12%)
21–30 min	10	7 (70%)	3 (30%)
>30 min	12	3 (25%)	9 (75%)
**Exercise intervention**			
Total time			
≤10 min	8	3 (38%)	5 (62%)
11–20 min	24	17 (71%)	7 (29%)
21–30 min	20	13 (65%)	7 (35%)
>30 min	5	2 (40%)	3 (60%)
Type			
Aerobic exercise	43	28 (65%)	15 (35%)
Combined exercise	14	7 (50%)	7 (50%)
Modality			
Running	12	7 (58%)	5 (42%)
Cycling	28	19 (69%)	9 (31%)
Circuit training	14	7 (50%)	7 (50%)
Boxing	3	2 (67%)	1 (33%)
Rest interval			
Active	30	21 (70%)	9 (30%)
Passive	27	14 (52%)	13 (48%)
Work recovery ratio			
>1	25	16 (64%)	9 (36%)
<1	13	7 (54%)	6 (46%)
=1	19	12 (63%)	7 (37%)
Intensity			
Maximal	21	12 (57%)	9 (43%)
Submaximal	36	23 (64%)	13 (36%)
**Sample and study characteristics**			
Age			
≤18 years	13	7 (54%)	6 (46%)
19~40 years	40	27 (68%)	13 (32%)
>40 years	4	1 (25%)	3 (75%)
Gender			
>50% male	33	22 (67%)	11 (33%)
<50% male	23	12 (52%)	11 (48%)
Equal	1	1 (50%)	1 (50%)
Fitness level			
Fit	53	33 (62%)	20 (38%)
Sedentary	4	2 (50%)	2 (50%)
Study Design			
Within-subject	38	25 (66%)	13 (34%)
Between subject	19	10 (53%)	9 (47%)
Comparator			
Active control	2	1 (50%)	1 (50%)
Passive control	55	34 (62%)	21 (38%)

Note: EF = executive function.

## Data Availability

All datasets generated for this research are included in this published article.
